# Mr. Harrison's Case of Dolor Faciei

**Published:** 1803-11-01

**Authors:** John Ring

**Affiliations:** New Street, Hanover Square


### *To the Editors of the Medical and Physical Journal.*

GENTLEMEN,

THE following Case was communicated to me by Mr. Carter, who had been a witness to the sufferings of the patient, and the success of the operation, when he was with Mr. Harrison. It is extracted from a letter of Mr. Harrison, addressed to Mr. Carter, when attending the hospitals in London; and was drawn up at my request, for insertion in your valuable Journal.

"Mr. William Dixon, of the Green Yew, Winster, of a strong muscular make, robust constitution, and about sixty years of age, became affected with a slight pain in the left side of his face about six years ago; which gradually increased, and assumed all the characters of the dolor faciei, as described by Fothergill, and others, in its most distressing state.

"It is, therefore, unnecessary to enter into a minute detail of the symptoms; suffice it to say, that the most trifling exertion, such as pulling the bed-clothes up, or turning in bed, would excite the most excruciating agony.

"He was often unable to bear shaving on that side of his face, until he had sat with his legs in warm water for a considerable time. He was not able to eat a bit of bread till after he had undergone the operation for his disease.

"I first saw him on the 15th of April, 1802. Pain and misery were, perhaps, never more strongly pictured on any living countenance than on his. He had been under the care of several respectable practitioners; taken cicuta, mercury, gum guaiacum, bark, &c.; and applied leeches, blisters, and various other stimulants, without the least alleviation of his sufferings.

"I was decidedly of opinion, that nothing but the division of the infraorbital nerve would be likely to relieve this dreadful complaint; and he submitted to the operation with the greatest fortitude; assuring me, that nothing could add to the tortures he already endured.

"The incision was made of a semilunar shape, an inch in length, through the integuments of the cheek, immediately below the infra-orbital foramen; through which the nerve passes from the fifth pair, to be distributed to the face, and in some instances sends one or more branches to communicate with the portio dura of the seventh pair.

"Notwithstanding the knife apparently reached the bone, bone, the whole length of the incision, yet I was convinced the nerve was not divided, from his not complaining, nor even distorting a muscle. I therefore introduced the fore-finger of my left hand into the wound; and feeling the hole through which the nerve came with a sharp pointed scalpel, made sure that nothing remained undivided between it and the bone.

"The pain this produced was so severe, that it forced a shriek from the most determined fortitude I ever saw; and, instantly applying a finger to the left angle of his upper lip, he exclaimed it was quite numbed.

"After the hæmorrhage had ceased, the edges of the wound were drawn together by means of sticking-plaster, and dressed superficially; after which he ate and drank with a degree of pleasure, which I shall never forget.

"The wound healed by the first intention; and, from the moment when the nerve was divided, I have the satisfaction to add, he has never had the least return of his complaint.

"From the concealed and deep situation of the nerve, immediately after it issues from the infra-orbital foramen of the maxilla superior, and from what happened in this case, I am confident, that in performing the operation, simple as it is, the nerve may easily escape the knife; and unless it be detached from its connection with the sensorium commune, no relief can be expected.

*Yelverton,* "I am, &c.

*June* 6, 1803. "WILLIAM HARRISON."

This case deserves attention; and, if surgeons will observe the hint here given by Mr. Harrison, time will shew, whether the operation so frequently fails from a re-union of the nerve taking place, or from its not being divided.

In the last number of your Journal is a letter from a gentleman, whom I am under the necessity of calling the late Mr. Dennett; in which are expressed two opinions, totally differing from those commonly entertained.

The first is, that in inoculation, incision produces less inflammation than puncture; the second, that dry variolous matter produces a milder disease than fluid.

Were the latter opinion well founded, I apprehend the discovery would soon have been made by every other considerable inoculator. To that character, with respect to the small-pox, I never had any pretension; but having inoculated some hundreds with dry matter, as well as with fluid, I never could distinguish any difference in their effects.

 With

With regard to vaccine inoculation, I never had the least idea, that in publishing an account of his mode of preserving and inserting the virus, Mr. Dennett meant any thing more than to ensure infection. When I read, in your Journal for 1801, that his manner of inserting it was by making three or four incisions or scratches across the arm, about one-fifth of an inch long, and only half a line apart, I dreaded consequences far worse than those which Mr. Dennett has recorded, as arising from puncture.

Such consequences, I have great reason to believe, no puncture, or vaccine vesicle excited by puncture, ever occasioned, or ever will occasion, unless the arm be irritated by some other cause. It is, however, not unnecessary to observe, that even the puncture ought to be small and superficial. For ensuring infection by this mode, fluid matter is preferable to dry; and from much experience, I confidently affirm, that it is not more virulent, as Mr. Dennett supposed.

Much worse cases than that recorded by Mr. Dennett, occurred at Clapham; partly, if not entirely, in consequence of incisions; of which your Journal bears ample testimony.

Had not the case in question been more severe than common, no practitioner in the neighbourhood would have been consulted. It would be easy to mention several worse cases occasioned by incision; but over these, hoping, and believing, that the practice is on the decline, I wish to draw a veil.

Many practitioners have discontinued incisions in consequence of my request; and have confessed, what indeed is apparent to others as well as to themselves, that their practice is much more successful than it was before.

Mr. Dennett acknowledges, that the blood is not infectious, yet disapproves of inoculating more than one person with one vaccinator. It was not necessary for him to do this; nor is it necessary for me; but it may be necessary for practitioners, in a situation remote from the metropolis, when the small-pox is raging on every side, and they can procure but a scanty supply of matter. In such cases, every one must use his own discretion.

I have exerted my utmost efforts to prevent that necessity, by facilitating the conveyance of matter on very small instruments; twenty or thirty of which may be contained in a single quill. Hence there is less occasion to inoculate more than one person with one instrument.

The demand for cow-pock matter is still considerable; but but the sources of supply are much more numerous. The Royal Jennerian Society has already opened thirteen stations, besides a central house. At all these places, matter is distributed free of expence.

I am, &c.

*New Street, Hanover Square,*
*October* 12, 1803.

JOHN RING.

